# Optimizing flue-cured tobacco planting patterns: enhanced rhizosphere nutrient availability and microbial community dynamics

**DOI:** 10.3389/fmicb.2026.1735540

**Published:** 2026-03-13

**Authors:** Furen Sun, Mouzhi Yuan, Chengyue Liao, Yaqian Sun, Linyi Yu, Yifeng Zhuo, Yinhe Peng, Xiaoming Tang, Qiang Zeng, Jiaqing Song, Xuan Tao, Qiang Li, Minggang Chen, Yiyang Zhang

**Affiliations:** 1 College of Agriculture, Hunan Agricultural University, Changsha, Hunan, China; 2 Zhangjiajie Tobacco Company, Hunan Provincial Tobacco Company, Zhangjiajie, Hunan, China; 3 Raw Material Procurement Center, China Tobacco Hunan Industrial Co., Ltd., Changsha, Hunan, China

**Keywords:** hizosphere microbiome, carbon-nitrogen metabolism, continuous cropping obstacles, crop rotation, soil

## Abstract

**Introduction:**

Continuous monoculture of flue-cured tobacco causes soil degradation and microbial dysbiosis. While crop rotation can alleviate these obstacles, how different cropping patterns regulate soil carbon (C) and nitrogen (N) metabolic functions remains unclear.

**Methods:**

A four-year field experiment compared tobacco monoculture (CK), tobacco–maize rotation (TM), tobacco–rice rotation (TR), and tobacco–sweet potato intercropping (TP). Soil physicochemical properties, enzyme activities, metagenomic sequencing, and microbial network analysis were integrated.

**Results:**

TR significantly improved soil health: pH (+6.6%), organic matter (+22.1%), and urease activity (+12.5%). It enriched beneficial microbes (*Pseudomonadota* +16.4%, *Mucoromycota* +327%) and upregulated C-cycle (*korA* +42.3%) and N-assimilation genes (*amoC* +460%), while suppressing denitrification (*nirK*). TM increased available P/K but enriched oligotrophic taxa and reduced sucrase activity. TP triggered pathogenic fungi (*Olpidium* +160%), depleted beneficial microbes, and broadly suppressed C/N metabolic genes (*cbbL* –94.5%, *nirS* –21.8%).

**Discussion:**

Cropping patterns differentially reshape microbial communities and metabolic functions, determining their efficacy against continuous cropping obstacles. TR establishes efficient C/N cycling with “high assimilation, low denitrification,” whereas TP induces pathogenic proliferation and metabolic suppression. This provides a functional framework for designing cropping systems to enhance soil health and tobacco productivity.

## Graphical abstract

**Figure:**
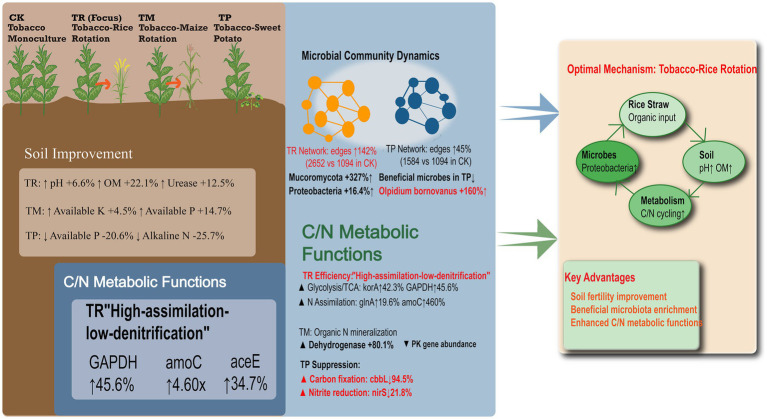
Cropping patterns reshape soil fertility and microbial communities. Pattern-specific differentiation in carbon and nitrogen metabolic functions among cropping patterns.

## 1 Introduction

Long-term continuous monoculture inevitably leads to soil nutrient imbalance and the accumulation of soil-borne pathogens and pests. These issues adversely affect plant health and plant yield, ultimately constraining sustainable agricultural development. As a significant economic crop in China, flue-cured tobacco (Nie and Li, 2020) faces specific challenges under long-term continuous cropping. Specifically, tobacco plants release root exudates with distinct compositions that significantly alter the composition and structure of the soil microbial community. This alteration modifies the fungal-to-bacterial ratio (Chen et al., 2025), increases the incidence of diseases and pests, and contributes to soil nutrient disorders (Hiremath et al., 2024).

Crop rotation and intercropping represent proven agricultural strategies for mitigating continuous cropping obstacles by improving soil nutrient status and enriching beneficial soil bacterial phyla (Tamburini et al., 2020).

Research on the application of rotation (e.g., with maize, rice, and sweet potato) and intercropping within flue-cured tobacco cropping systems has demonstrated significant progress. Li et al. (2024) reported that rice–lily rotation, compared to lily monoculture, significantly increased soil pH by 0.39 units, effectively alleviating soil acidification, and enhancing the abundance of beneficial fungal genera such as *Fusarium* and *Mortierella*. Dai et al. (2024) found that tobacco–radish intercropping significantly improved soil aggregate structure, enhanced cation exchange capacity, improved water-stable aggregate stability, and reduced disease incidence. Wang et al. (2024) demonstrated that tobacco–gromwell rotation increased soil organic matter (OM) by 17.38%, available phosphorus (AP) by 2.78%, and available potassium (AK) by 7.76%, while also enhancing sucrase (SC) and urease (URE) activities.

However, the majority of existing studies have focused on describing changes in microbial community composition, while a key scientific question remains unclear: how do different cropping patterns differentially alter the core metabolic functions of rhizosphere microorganisms, particularly those involved in carbon and nitrogen cycling processes directly related to soil health and crop nutrition? The limited understanding of this functional mechanism constrains our ability to precisely design cropping systems from a microbial-function perspective.

Based on this perspective, we propose the following core hypothesis: in paddy–upland tobacco planting systems, different rotation or intercropping patterns will, through the reciprocal influences of root exudates among crops or via rhizosphere resource inputs (e.g., straw return), selectively enrich microbial taxa with specific metabolic functions. This process leads to pattern-specific variations in gene abundance and activity within key pathways involved in soil carbon and nitrogen transformation. This functional divergence may explain the differential effectiveness of various patterns in alleviating continuous cropping obstacles.

## 2 Materials and methods

### 2.1 Experimental design

A long-term field experiment was established in 2020 at a single site in Yanzi Village (29°25′57.9″N, 111°8′2.2″E), Lingxi Town, Cili County, Zhangjiajie City, Hunan Province, China. This region experiences a subtropical monsoon climate with a mean annual temperature of 16.8 °C, an average annual precipitation of 1,465.2 mm, and a mean annual sunshine duration of 1,562.7 h. The soil at this location is uniformly classified as “Paddy soil” (Haplic Stagnosol, World Reference Base for Soil Resources; or Typic Haplaquept, United States Department of Agriculture (USDA) Soil Taxonomy). To investigate the effects of different cropping patterns under consistent edaphic and climatic conditions, four treatments were established in a randomized complete block design with three replicates, resulting in a total of 12 plots (each 10 m × 13 m): (1) flue-cured tobacco monoculture (CK); (2) tobacco–maize rotation (TM); (3) tobacco–rice rotation (TR); and (4) tobacco–sweet potato intercropping (TP). Soil sampling and analyses presented in this study were conducted in 2024, after four consecutive years of treatment application, to assess their medium-term effects on soil properties and microbial communities.

### 2.2 Soil sample collection

Soil samples were taken based on the flue-cured tobacco season (July) and the post-tobacco crop season (November), corresponding to the maturity and harvest time of the subsequent crops, respectively. Rhizosphere soil was collected from three randomly selected plants per plot. Plants were carefully uprooted, and the soil tightly adhering to the roots after gentle shaking was defined as the rhizosphere soil. This soil was brushed off using a sterile brush. The rhizosphere soil from the three plants within the same plot was combined and thoroughly mixed to form one composite sample per plot, resulting in a total of approximately 1 kg of soil. After removing debris such as litter and gravel, each composite soil sample was divided into two portions. One portion was sieved (2-mm mesh), and the subsamples were placed into 50-mL and 100-mL screw-cap centrifuge tubes. These tubes were stored in insulated containers with dry ice packs, transported to the laboratory, and subsequently stored at −80 °C. The samples in the 50-mL tubes were promptly sent to Sanshu Biotech Co., Ltd. (Nantong, Jiangsu, China) for metagenomic sequencing. The samples in the 100-mL tubes were used for determining key soil enzyme activities, including urease, sucrase, acid phosphatase (ACP), and catalase (CAT).

The remaining portion of the soil sample was air-dried naturally at room temperature, sieved (0.15-mm or 2-mm mesh; please specify mesh size if known), and used for the analysis of routine soil physicochemical properties (Gong et al., 2024).

### 2.3 Analytical methods

#### 2.3.1 Determination of soil chemical properties

Soil pH was determined potentiometrically. OM content was measured using the potassium dichromate oxidation method with oil-bath heating. Alkali-hydrolyzable nitrogen (AN) was quantified by the alkaline hydrolysis diffusion method. AK was extracted with ammonium acetate and determined by atomic absorption spectrometry. AP was extracted with sodium bicarbonate and quantified using the molybdenum–antimony anti-spectrophotometric method (Soil Science Society of America and Weaver, 1994). Total nitrogen (TN) was determined by the Kjeldahl digestion method. Total phosphorus (TP) was measured using the NaOH fusion-molybdenum antimony anti colorimetric method. Total potassium (TK) was determined by NaOH fusion followed by flame photometry.

#### 2.3.2 Determination of soil enzyme activities

For each enzyme assay, 5 g of fresh soil was used. ACP activity was determined using the p-nitrophenol colorimetric method with modified universal buffer (pH 6.5). Sucrase (SC) activity was measured by the 3,5-dinitrosalicylic acid (DNS) colorimetric method in phosphate buffer (pH 5.5). Urease (URE) activity was assayed via indophenol blue colorimetry using the urea solution in citrate buffer (pH 6.7). Catalase (CAT) activity was determined by potassium permanganate titration. Protease (NEU) activity was measured using the Folin reagent colorimetric method (Liang-liang et al., 2023). All assays were performed in triplicate.

#### 2.3.3 Soil DNA extraction and sequencing

Soil DNA was extracted using the cetyltrimethylammonium bromide (CTAB) method. The quality and concentration of the extracted DNA were assessed using 1% agarose gel electrophoresis and an ND-1000 UV–Vis spectrophotometer. DNA samples were then sent to Sanshu Biotech Co., Ltd. (Nantong, Jiangsu, China) for metagenomic sequencing.

Sequencing libraries were constructed using the NEBNext® Ultra™ DNA Library Prep Kit (New England Biolabs, NEB, USA). Briefly, the DNA samples were randomly fragmented into approximately 350-bp fragments using ultrasonication. The library preparation process included end repair, A-tailing, adapter ligation, purification, and PCR amplification. Library concentrations were accurately quantified using quantitative PCR (qPCR).

Metagenomic sequencing was performed on the Illumina HiSeq X Ten platform. Raw sequencing data were processed using Trimmomatic software to obtain high-quality clean data. Sequences were then assembled using MEGAHIT, and the sequences shorter than 500 bp were filtered out. Open reading frames (ORFs) were predicted using MetaGeneMark, and redundancy was reduced with CD-HIT using a 95%similarity threshold and a 90%coverage threshold. A final gene catalog was generated from non-redundant genes.

All ORFs were annotated using DIAMOND and HMMER against the Kyoto Encyclopedia of Genes and Genomes (KEGG) and Carbohydrate-Active Enzymes (CAZy) databases (Yang et al., 2021).

#### 2.3.4 Metagenomic data processing and statistical analysis

One-way analysis of variance (ANOVA) was performed using SPSS version 22.0 on flue-cured tobacco parameters and soil chemical properties. Figures were generated using Origin 2023. Spearman correlation analysis was performed to assess the relationships between the relative abundance of dominant soil microbial species and soil chemical properties.

Molecular ecological networks were constructed using the Hmisc R package, retaining significant species-level correlations (*p* < 0.05) with |*R*| > 0.6 based on Spearman correlation analysis. Network visualization was performed using Gephi.

## 3 Results

### 3.1 Differences in rhizosphere soil chemical properties among cropping patterns

Different cropping patterns significantly influenced the chemical properties of rhizosphere soil (Table 1). Compared to the flue-cured tobacco monoculture (CK), all treatments resulted in increases in soil pH and total potassium (TK) content. The TR treatment (tobacco-rice rotation) significantly increased soil pH and OM content compared to CK (*p* < 0.05), by 6.6 and 22.1%, respectively. The TM treatment (tobacco-maize rotation) significantly enhanced the contents of soil AP (*p* < 0.01) and AK (*p* < 0.01), with increases of 14.7 and 4.5%, respectively. In contrast, the TP treatment (tobacco-sweet potato intercropping) had significant negative effects on the majority of soil nutrient indicators. Its soil total phosphorus (TP; *p* < 0.01), AP (*p* < 0.01), and available nitrogen (AN; *p* < 0.01) contents were all significantly lower than those in CK, with the largest decrease observed for AN (−25.6%). Overall, the TR and TM patterns demonstrated greater potential for improving the chemical environment of the rhizosphere soil.

**Table 1:** Differences in rhizosphere soil chemical properties among cropping patterns.

Treatment	pH	Organic matter (g/kg)	Total nitrogen (%)	Total phosphorus (g/kg)	Total potassium (g/kg)	Available phosphorus (mg/kg)	Available potassium (mg/kg)	Available nitrogen (mg/kg)
CK	6.36 ± 0.05	28.67 ± 1.33	0.205 ± 0.01	0.681 ± 0.011	22.37 ± 0.23	22.67 ± 0.61	227.22 ± 0.55	166.45 ± 0.37
TM	6.52 ± 0.04*	30.46 ± 0.9	0.194 ± 0.007	0.602 ± 0.013**	24 ± 0.2**	26 ± 0.5**	237.42 ± 0.48**	130.62 ± 0.3**
TP	6.47 ± 0.16	31.76 ± 0.25	0.194 ± 0.015	0.564 ± 0.008**	23 ± 0.2*	18 ± 0.2**	200.65 ± 0.8**	123.78 ± 0.11**
TR	6.78 ± 0.04**	35 ± 0.2*	0.213 ± 0.009	0.631 ± 0.002*	23.5 ± 0.2**	24 ± 0.2	220.4 ± 1.9*	165.49 ± 0.38*

*p < 0.05; **p < 0.01, compared with the CK treatment.

CK, tobacco monoculture; TR, tobacco–rice rotation; TP, tobacco–sweet potato intercropping; TM, tobacco–maize rotation.

Compared to flue-cured tobacco monoculture (CK) (Table 2), the effects of different treatments on soil enzyme activities varied, revealing complex regulatory patterns. The TR treatment significantly enhanced the activities of soil SC (*p* < 0.01) and URE (*p* < 0.01), which increased by 29.7 and 12.5%, respectively, compared to CK. It also significantly reduced the activities of ACP and neutral NEU.

**Table 2:** Variations in rhizosphere soil enzyme activities among cropping patterns.

Treatment	Catalase activity (μmol/g)	Acid phosphatase activity (μmol/g)	Dehydrogenase activity (μg/g)	Neutral protease activity (mg/g)	Sucrase activity (mg/g)	Urease activity (μg/g)
CK	70.98 ± 0.15	33 ± 0.17	70.49 ± 0.39	0.304 ± 0.007	12.27 ± 0.16	175.33 ± 0.59
TM	37.58 ± 0.39**	27.7 ± 0.49**	126.95 ± 0.06**	0.154 ± 0.01**	10.1 ± 0.1**	170.14 ± 0.21**
TP	59.87 ± 0.13**	30.09 ± 0.16**	65.49 ± 0.43**	0.32 ± 0.016	10.21 ± 0.2**	191.36 ± 0.28**
TR	69.05 ± 0.08**	25.94 ± 0.07**	78.91 ± 0.1**	0.153 ± 0.009**	15.92 ± 0.06**	197.33 ± 0.52**

*p < 0.05; **p < 0.01, compared with the CK treatment.

CK, tobacco monoculture; TR, tobacco–rice rotation; TP, tobacco–sweet potato intercropping; TM, tobacco–maize rotation.

The TM treatment generally suppressed the majority of enzyme activities, with CAT, ACP, neutral NEU, and SC activities being significantly lower than those in CK (*p* < 0.01). Among these, CAT activity (37.58 ± 0.39 μmol/g) decreased most substantially, by 47.1%. However, dehydrogenase (DHA) activity in this treatment (126.95 ± 0.06 μg/g) was significantly enhanced by 80.1%, representing the highest value among all treatments.

The TP treatment significantly increased URE activity (191.36 ± 0.28 μg/g; *p* < 0.01) and significantly decreased ACP and SC activities, while no significant effect was observed on NEU activity.

### 3.2 Differences in rhizosphere soil microorganisms under different planting patterns

#### 3.2.1 Soil microbial community composition

**Figure 1:**
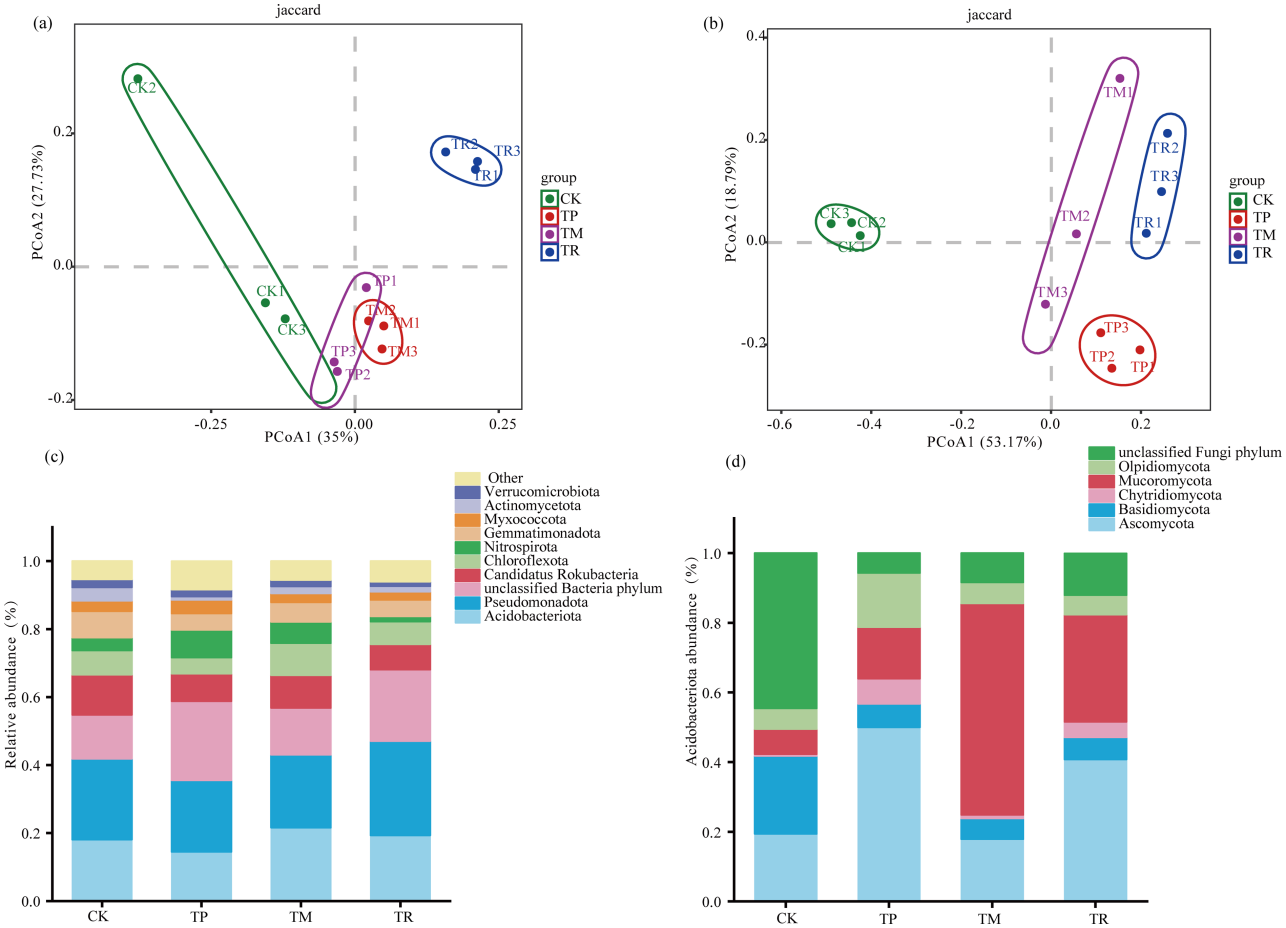
Composition and β-diversity of soil microbial communities. **(a)** Principal coordinate analysis (PCoA) of bacterial communities based on Jaccard distance. **(b)** PCoA of fungal communities based on Jaccard distance. **(c)** Relative abundance of dominant bacterial phyla. **(d)** Relative abundance of dominant fungal phyla.

Principal coordinate analysis (PCoA) based on Jaccard distance at the genus level revealed distinct clustering of soil microbial communities among treatments (Figures 1A,B). For bacterial communities, PCoA axis1 and PCoA axis 2 accounted for 35.00 and 27.73% of the total variation, respectively (Figure 1A). For fungal communities, PCoA axis 1 and PCoA axis 2 explained 53.17 and 18.79% of the variation, respectively (Figure 1B). The clear separation of samples by treatment indicated significant differences in both bacterial and fungal community composition.

At the phylum level (Figure 1C), *Acidobacteriota* and *Pseudomonadota* predominated across all treatments, alongside unclassified bacteria. Compared to the control (CK), the TR treatment exhibited higher relative abundances of both *Acidobacteriota* and *Pseudomonadota*. Notably, *Pseudomonadota* abundance in TR was significantly higher than in all other treatments, showing a 16.4% increase compared to CK. Conversely, TR showed significantly lower abundances of *Chloroflexota* and *Actinomycetota* compared to CK. In the TM treatment, *Acidobacteriota* and *Chloroflexota* abundances were significantly higher than in CK by 19.3 and 32.8%, respectively. The TP displayed a significantly higher relative abundance of *Nitrospirota* than other patterns. However, TP also had significantly lower abundances of *Acidobacteriota*, *Pseudomonadota*, *Chloroflexota*, and *Actinomycetota* compared to CK, with *Chloroflexota* and *Actinomycetota* reduced by 33.1 and 75.3%, respectively.

Among the top 10 fungal phyla (Figure 1D), *Mucoromycota* and *Ascomycota* were the most abundant, excluding unclassified fungi. The TP treatment showed significantly higher relative abundances of *Ascomycota*, *Chytridiomycota*, and *Olpidiomycota* compared to the other three treatments. Importantly, the TR treatment significantly increased the abundances of *Mucoromycota* and *Ascomycota* by 327 and 109%, respectively, compared to CK.

**Figure 2:**
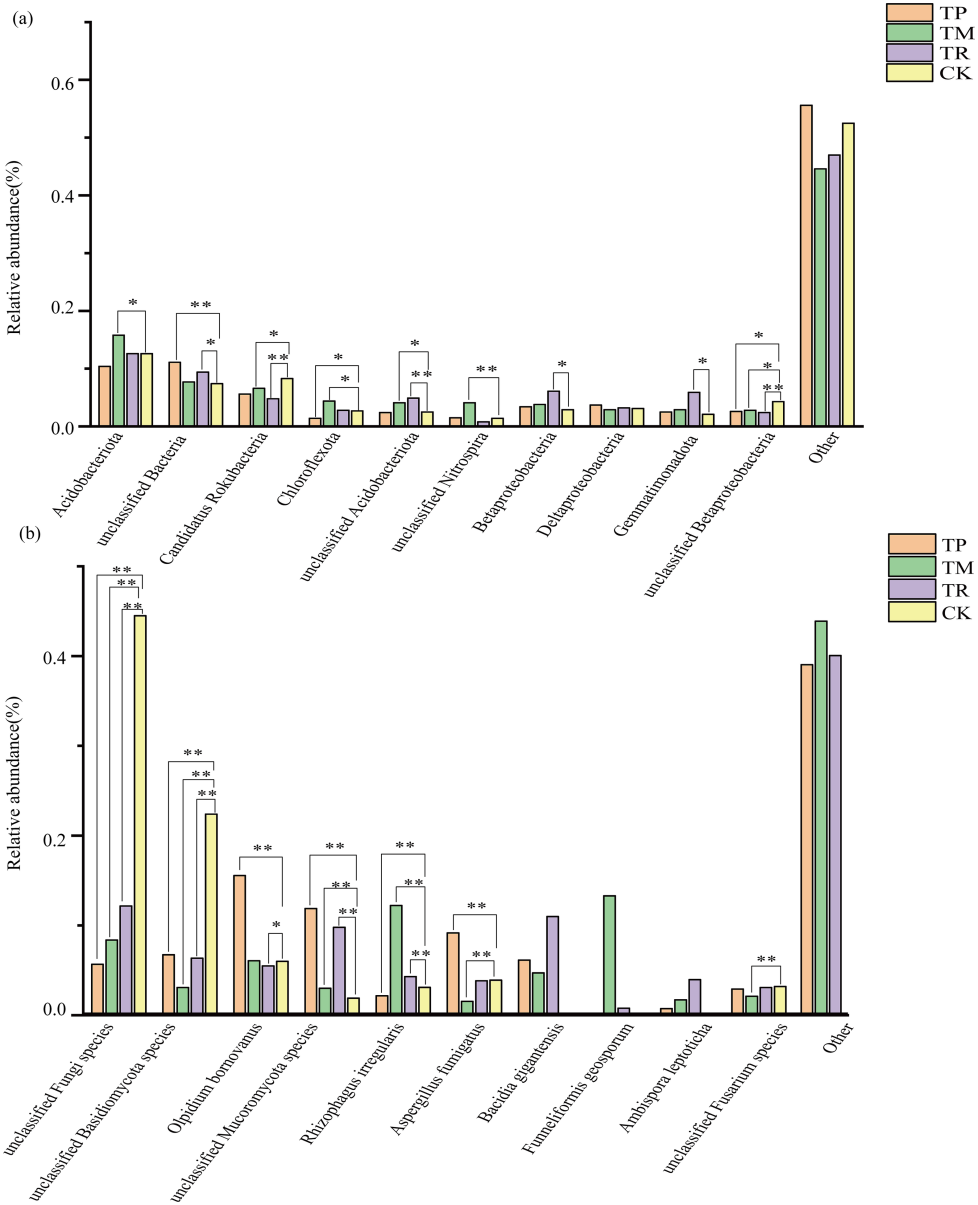
Relative abundance of soil microorganisms at the species level: **(a)** relative abundance of bacterial communities at the species level and **(b)** relative abundance of fungal communities at the species level. *p < 0.05; **p < 0.01.

The relative abundance of soil microbial communities at the species level under different planting patterns is shown in Figure 2. Significant differences (*p* < 0.05), were observed in bacterial and fungal community composition at the species level among the four planting patterns. The fungal community structure exhibited greater sensitivity to planting patterns. In the TM treatment, the relative abundances of *Betaproteobacteria* bacterium and *Chloroflexota* bacterium were higher, showing significant increases of 28.4 and 18.3%, respectively, compared to CK. The relative abundance of *Beta Pseudomonadota* bacterium in the TR treatment was 100% higher than in CK (Figure 2b). At the fungal species level, the relative abundances of *Olpidium bornovanus* and *Aspergillus fumigatus* in the TP treatment were the highest among the four treatments, with significant increases of 160 and 135%, respectively, compared to CK (Figure 2b).

#### 3.2.2 Properties of soil microbial molecular networks

**Figure 3:**
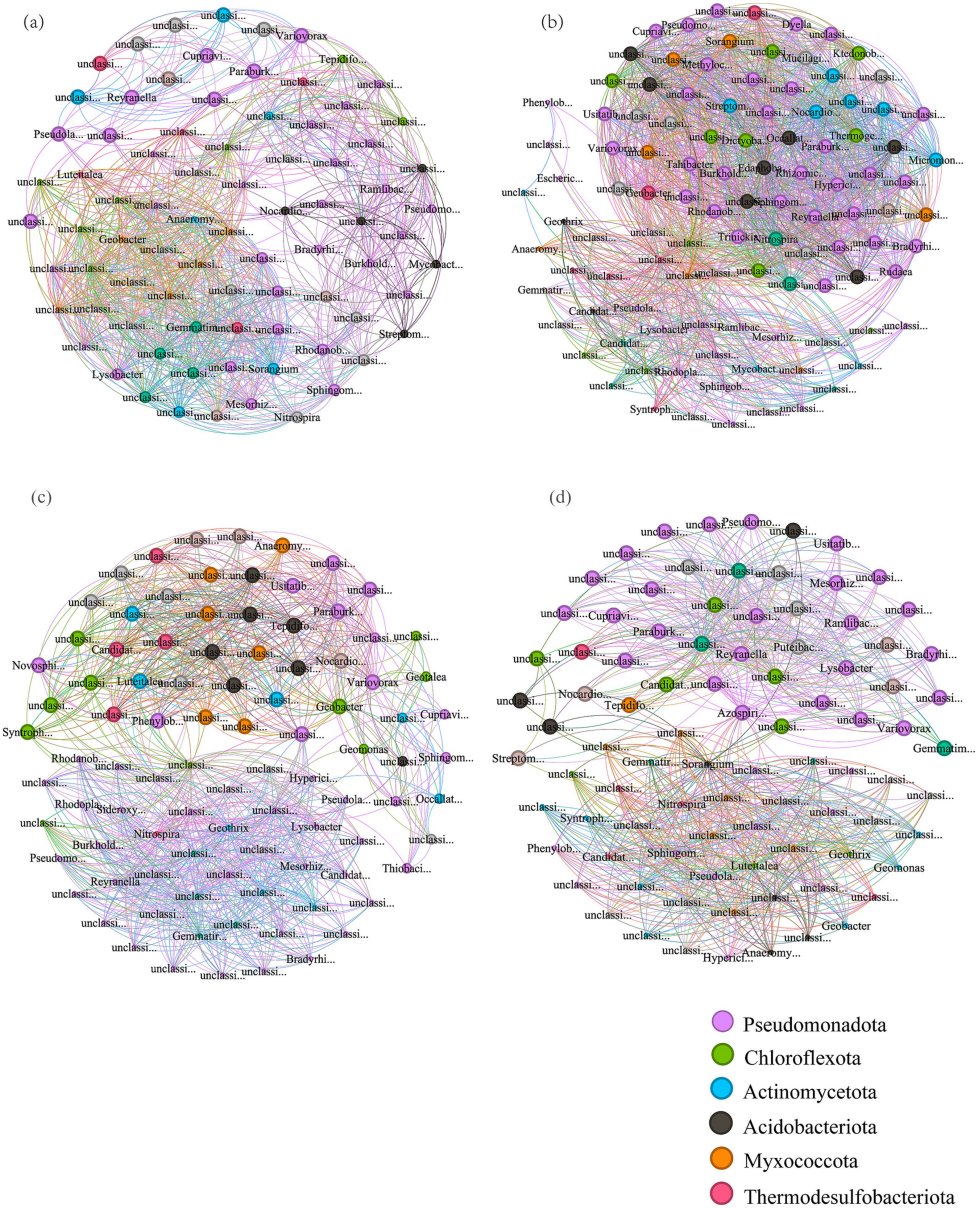
Co-occurrence networks of soil bacterial communities. **(a)** Network for the CK treatment. **(b)** Network for the TR treatment. **(c)** Network for the TP treatment. **(d)** Network for the TM treatment (CK, tobacco monoculture; TR, tobacco–rice rotation; TP, tobacco–sweet potato intercropping; TM, tobacco–maize rotation).

Microbial co-occurrence networks were constructed at the genus level (relative abundance > 0.005%). Nodes were colored by phylum-level taxonomy in Figures 3a–d, corresponding to CK, TR, TP, and TM treatments, respectively. The networks primarily consisted of *Pseudomonadota*, *Chloroflexota*, *Actinomycetota*, *Acidobacteriota*, *Myxococcota*, and *Thermodesulfobacteriota*. Topological properties are presented in Table 3. The results indicate that efficient cropping patterns promoted microbial interactions, resulting in more complex networks. Positive correlations dominated microbial interactions across all treatments. Node numbers ranked as TR > TP > TM > CK. Furthermore, TR, TP, and TM treatments showed higher node counts, edge numbers, average degrees, and graph densities than CK. Among all treatments, TR exhibited the highest node count (107), edge number (2652), and average degree (24.785), indicating the most complex network. Specifically, TR increased the node count by 32.1% and the edge number by 142.4% relative to CK.

**Table 3:** Topological properties of soil bacterial co-occurrence networks.

Treatments	Total nodes	Total links	Average degree	Graph density	Modularity
CK	81	1,094	13.506	0.169	0.384
TR	107	2,652	24.785	0.234	0.298
TP	94	1,584	16.851	0.181	0.48
TM	90	1,625	18.056	0.203	0.492

CK, tobacco monoculture; TR, tobacco–rice rotation; TP, tobacco–sweet potato intercropping; TM, tobacco–maize rotation.

#### 3.2.3 Relationships between soil microbial communities and soil chemical properties

**Figure 4:**
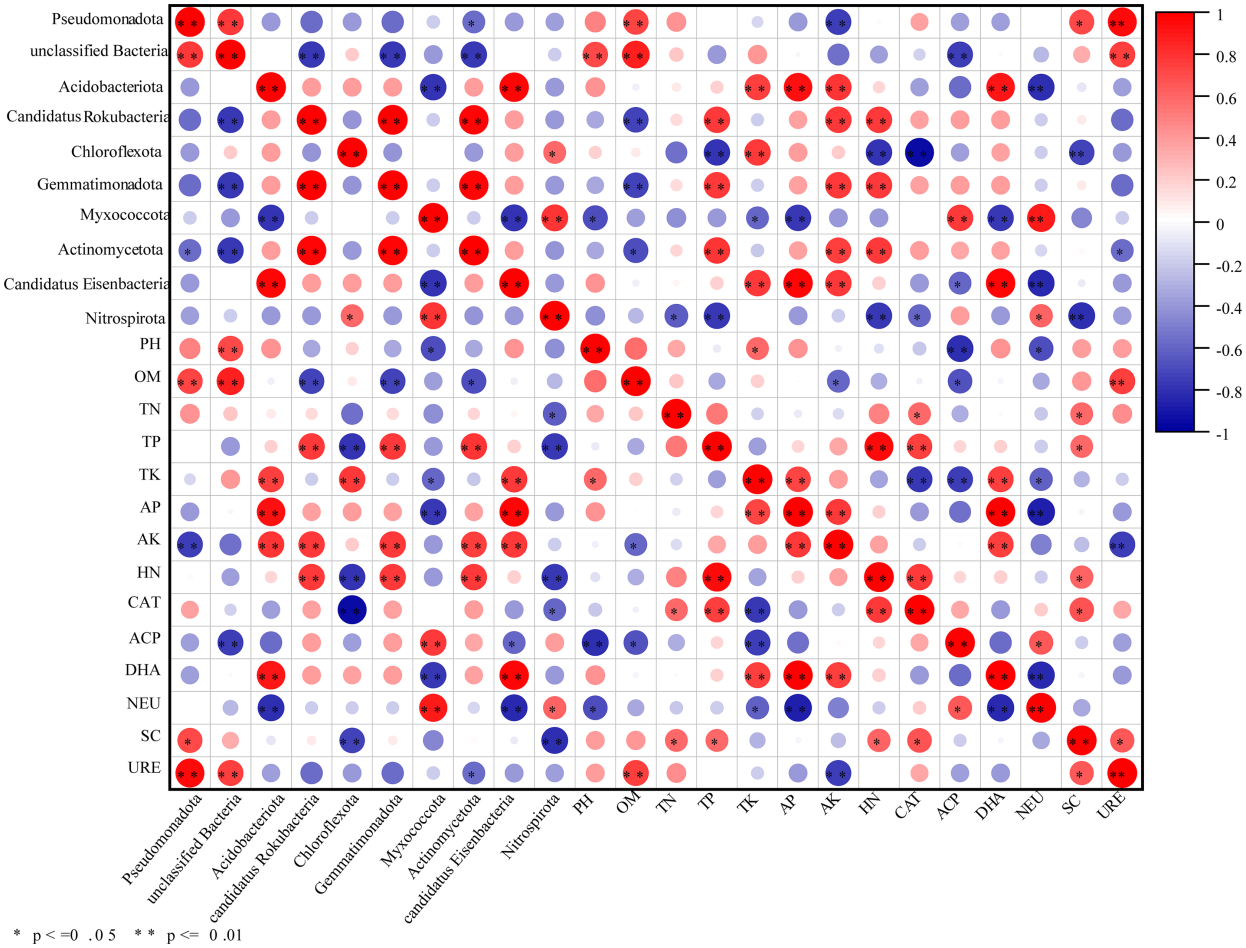
Spearman correlation analysis between soil physicochemical properties, enzyme activities, and the relative abundance of dominant bacterial phyla. OM, AN, AP, AK, SC, CAT, ACP, DHA, NEU, and URE represent organic matter, available nitrogen, available phosphorus, available potassium, sucrase, catalase, acid phosphatase, dehydrogenase, neutral protease, and urease, respectively.

Correlation analysis between the top 10 bacterial phyla and soil environmental factors is shown in Figure 4. Soil OM emerged as a key factor explaining the microbial community variation, associated with microbial adaptations in nutrient-rich environments. *Actinomycetota*—dominant in OM decomposition and antimicrobial substance secretion—showed significant positive correlations with TN, AK, and TP. *Pseudomonadota* exhibited significant positive correlations with OM, URE, and SC. *Acidobacteriota* displayed significant positive correlations with TK, AP, AK, and DHA. *Nitrospirota* were significantly negatively correlated with TN, SC, and TP. *Chloroflexota* showed a significant positive correlation with TK but significant negative correlations with SC, CAT, and TN.

#### 3.2.4 Functional diversity of soil microbial communities

**Figure 5:**
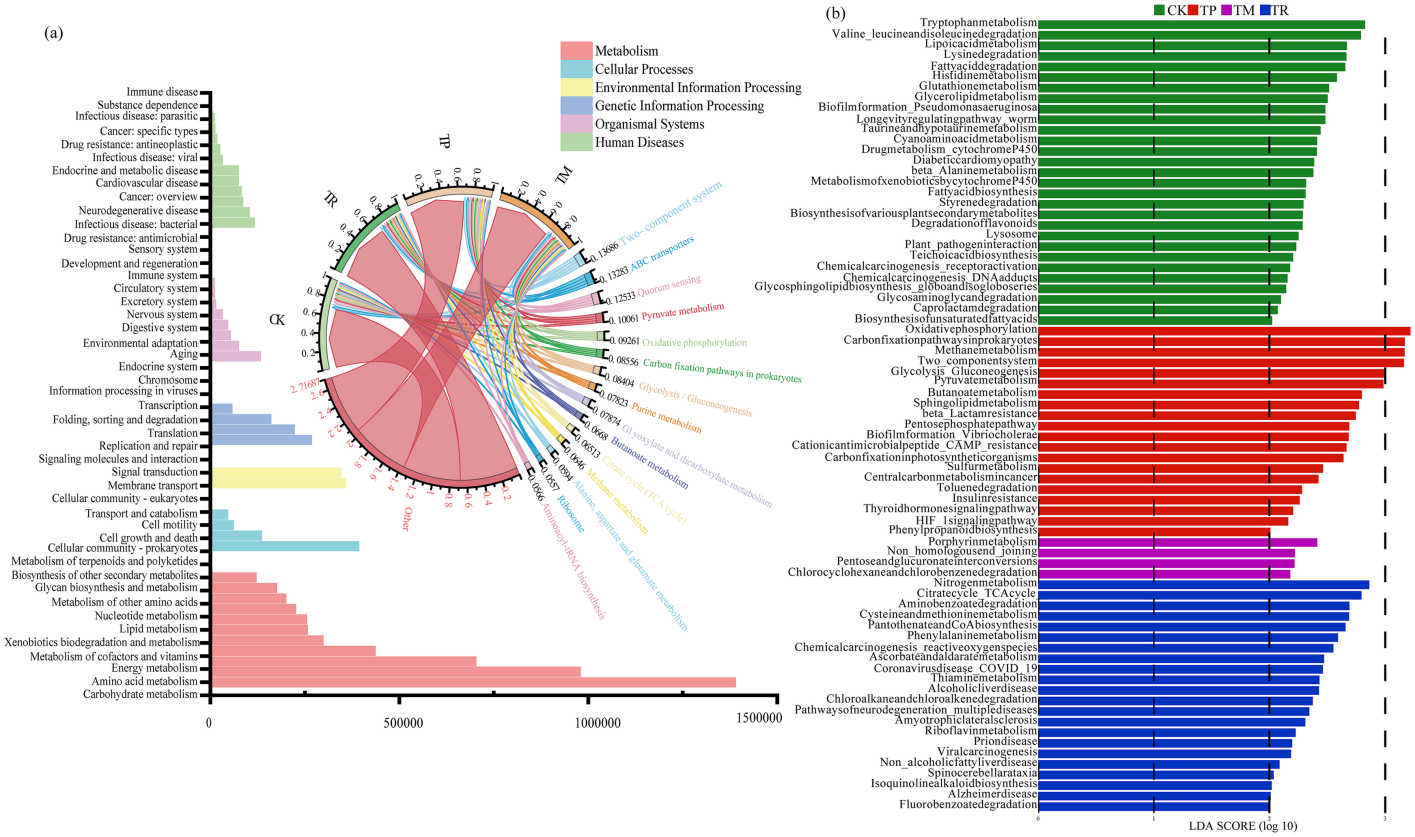
Functional profiling and differentially enriched pathways of soil microbial communities based on KEGG annotation. **(a)** Functional classification across pathway levels; **(b)** LEfSe (LDA effect size) analysis of differentially enriched KEGG pathways at level 3 (KEGG, Kyoto Encyclopedia of Genes and Genomes).

As shown in Figure 5a, the “Metabolism” category exhibited the highest gene abundance at pathway level 1 across all treatments. At level 2, this category primarily included the metabolism of cofactors and vitamins, global and overview maps, amino acid metabolism, carbohydrate metabolism, nucleotide metabolism, xenobiotics biodegradation and metabolism, and energy metabolism. At level 3, higher gene abundances were observed for glycolysis/gluconeogenesis, ABC transporters, pyruvate metabolism, and oxidative phosphorylation across treatments.

Linear discriminant analysis Effect Size (LEfSe) analysis based on linear discriminant analysis (LDA) at pathway level 3 (Figure 5b) revealed functional differences among treatments. CK showed significant enrichment in fundamental amino acid metabolism pathways: tryptophan metabolism, valine/leucine/isoleucine degradation, and lysine degradation. TP exhibited marked enrichment in carbohydrate metabolism pathways including oxidative phosphorylation, carbon fixation in prokaryotes, and methane metabolism. TR demonstrated significant enrichment in nitrogen metabolism, the tricarboxylic acid/citrate cycle (TCA cycle), and phenylalanine metabolism. TM had fewer enriched pathways, primarily comprising chlorocyclohexane/chlorobenzene degradation, pentose/glucuronate interconversions, and porphyrin metabolism.

**Figure 6:**
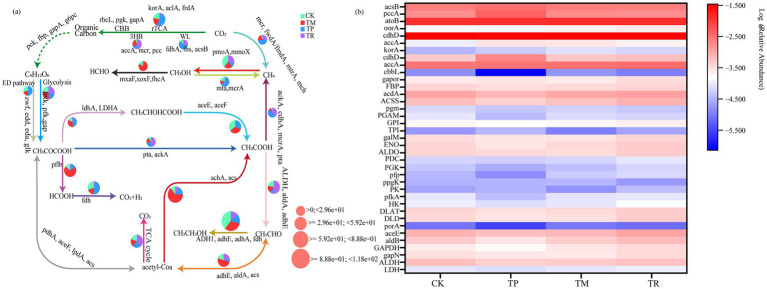
Genetic potential and key genes involved in soil carbon metabolism: **(a)** metabolic pathways regulated by carbon metabolism genes governing glycolysis and core three-carbon compound modules; **(b)** heatmap of carbon metabolism gene abundance.

Soil microorganisms exhibit diverse carbon metabolic pathways, primarily including the TCA cycle, Calvin cycle, dicarboxylate-hydroxybutyrate cycle, and 3-hydroxypropionate bicycle. Metagenomic analysis revealed that different treatments exerted specific effects on the genetic potential of glycolysis, the TCA cycle, and related branch metabolic pathways. Figure 6a illustrates the enzymes encoded by soil carbon metabolic genes involved in glycolysis and the core module of three-carbon compounds, along with their associated metabolic pathways. Key genes identified include those encoding glyceraldehyde-3-phosphate dehydrogenase (GAPDH), phosphoglycerate kinase (PGK), phosphoglycerate mutase (gpmB, gpmI, APGM), 2, 3-bisphosphoglycerate-dependent phosphoglycerate mutase (PGAM), enolase (ENO), pyruvate kinase (pyk), and pyruvate dehydrogenase (aceE, aceF, PDHA, PDHB) for glycolysis and gluconeogenesis pathways, as well as citrate synthase (gltA), and pyruvate decarboxylase (pdhA, pdhB, pdhC), and phosphoglycerate kinase (pgk) involved in the citrate cycle (TCA cycle). This indicates a robust genetic potential for glycolysis/gluconeogenesis pathways across treatments.

Figure 6b depicts the heatmap of carbon metabolism gene abundance, revealing that the TP treatment had significantly lower abundance of key glycolytic genes. Specifically, the abundance of the phosphofructokinase gene (pfkA) in TP (log_10_ = −4.38) was 9.1% lower than in CK (log_10_ = −4.02). Concurrently, the abundance of the pyruvate kinase gene (PK) was lowest in TM (log_10_ = −4.81). In contrast, TR exhibited a higher abundance of the GAPDH gene, which was 45.6% greater than in CK. TCA cycle genes showed divergent responses: TP had a lower abundance of DHA genes (e.g., DLD), potentially reducing cycle efficiency; TR showed an increase in the abundance of aceE by 34.7% relative to CK, which may facilitate pyruvate-to-acetyl-CoA conversion. In fermentative branches, TP showed reduced abundance of the lactate dehydrogenase gene by 19.3% versus CK, potentially inhibiting lactate synthesis; TR exhibited increased abundance of the pyruvate decarboxylase gene (PDC) by 51.3% versus CK, which could enhance ethanol fermentation. Notably, TP showed lower abundance of the ferredoxin oxidoreductase gene (porA) (−0.45 log_10_ units) but higher abundance of the glyceraldehyde-3-phosphate reductase gene (gapor), potentially enhancing NADPH generation; TM showed elevated abundance of the oorA gene, which may promote α-ketoglutarate metabolism. Carbon fixation/acetate metabolism responses were pronounced: TP showed a 94.5% decrease in the abundance of the Rubisco gene (cbbL) expression but increased abundance of accA, suggesting metabolic flux shift toward the propionate pathway; TR simultaneously showed higher abundance of acetate metabolism genes (ACSS +20.1%, acsB +7.5%) and the acetyl-CoA dehydrogenase gene (acdA), indicating the establishment of a complete acetate utilization pathway. Collectively, TP appeared to force metabolic rerouting by reducing the abundance of genes related to carbon fixation/glycolysis; TM modulated TCA branch points; TR established a pattern centered on pyruvate decarboxylation with high fermentative potential.

**Figure 7:**
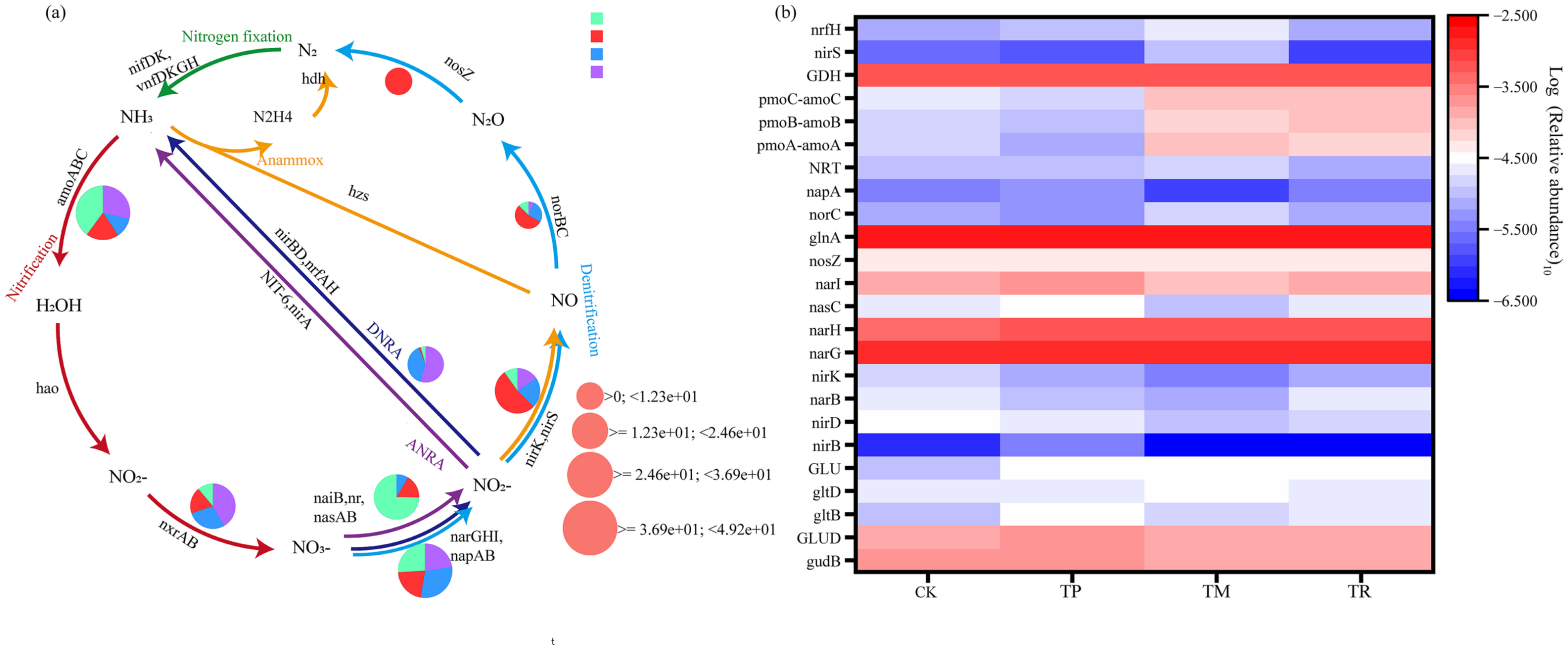
Genetic potential and key genes involved in soil nitrogen cycling. **(a)** Schematic diagram of soil nitrogen cycling pathways. **(b)** Heatmap of nitrogen metabolism gene abundance.

Metagenomic analysis of soil nitrogen metabolism genes revealed marked divergence in nitrogen cycling pathways among treatments (CK, TP, TM, TR), elucidating treatment-specific molecular response patterns. Soil nitrogen metabolism genes associated with the following processes were identified: assimilatory nitrate reduction, dissimilatory nitrate reduction, nitrification, denitrification, nitrogen fixation, and glutamate synthesis. Consequently, a schematic diagram of soil nitrogen cycling was constructed, annotating key enzymatic genes (Figure 7a). Glutamine synthetase (glnA), glutamate synthase (gltB, gltD), and glutamate dehydrogenase (GDH, gudB) were identified across treatments. Ammonia monooxygenase (pmoA-amoA, pmoB-amoB, pmoC-amoC) was associated with nitritation, while assimilatory nitrate reductase (narG, narH, narL) was linked to assimilatory nitrate reduction.

The nitrogen metabolism gene abundance heatmap (Figure 7b) revealed elevated abundances of glutamate synthesis genes (glnA, GDH, gudB) and nitrate reductase genes (narG, narH, narL), indicating a strong genetic potential for active ammonia assimilation and nitrate reduction. In CK, glnA and GDH showed high and coordinated abundance in ammonia assimilation, while TR exhibited peak abundance of both genes, supporting their role in glutamate synthesis. Concurrently, ammonia monooxygenase genes (pmoA-amoA, pmoB-amoB, pmoC-amoC) showed higher abundance in TR versus CK and TP, with pmoC-amoC exhibiting a 4.6-fold increase in abundance relative to CK, suggesting enhanced potential for nitrogen assimilation. During denitrification, nitrite reductase genes (nirK, nirS) showed lower abundance across treatments. TR displayed lower nirS and nirK abundance than CK and TM, with nirK being the lowest among treatments. This may lead to NO₂^−^accumulation and reduced potential for N₂O production, affecting the denitrification process.

## 4 Discussion

### 4.1 Effects of different cropping patterns on soil physicochemical properties and enzyme activities

Soil nutrients and enzyme activities are crucial for crop growth. Efficient cropping patterns such as rotation can alter the soil microenvironment and exert specific influences on soil nutrients and enzyme activities (Huang et al., 2019; Gu et al., 2021). Soil pH and TK under TR, TM, and TP were significantly higher than under CK, and soil OM was higher in TR and TP than in CK. Additionally, URE activity was significantly enhanced in TR and TP versus CK, consistent with previous research (Sun et al., 2020; Silva et al., 2022). This indicates that efficient cropping patterns (TR, TM, TP) differentially influence soil enzyme activity and improve soil fertility by increasing crop residue input or root exudates (Aleminew, 2024; Sun et al., 2024).

Regarding soil chemical properties, the TR pattern demonstrated the most significant improvements in soil pH, OM, and TK (Table 1). This relates directly to substantial organic carbon input from rice straw incorporation (Gaind and Singh, 2013), mitigating soil acidification from long-term tobacco cultivation (Kunkun et al., 2024), and promoting soil OM accumulation. The TM pattern significantly increased AP and AK, possibly related to maize root exudates and residue characteristics. Consistent with previous findings, rotating flue-cured tobacco with maize reduces nitrogen content while promoting root growth and enhancing P/K uptake (Yifan et al., 2024). In contrast, the TP pattern showed no distinct advantage in improving basic nutrients and even reduced TP, AP, and AN (Table 1).

Soil enzymes, as direct products of microbial activity and catalysts for biochemical processes, sensitively reflect cropping pattern effects. In this study, TR significantly increased SC and URE activities (Table 2). Alternating aerobic/anaerobic conditions in rotation create a dynamic environment conducive to ACP/URE activity (Majumder et al., 2022), closely linked to pH improvement (Olougou et al., 2024). The TM pattern exhibited significantly enhanced dehydrogenase (DHA) activity (+80.1%) CAT, ACP, neutral NEU, and SC activities (−47.1, −16.1%, −49.3%, and −17.7%, respectively). This apparent inconsistency with previous reports of increased URE/SC in TM (Ke et al., 2010) warrants attention. Although SC decreased, TM significantly increased soil OM (+10.7%) and DHA (+80.1%), directly reflecting microbial metabolic intensity. The TP pattern significantly increased URE activity and had no significant effect on neutral NEU activity, while it significantly decreased ACP and SC activities (Table 2). However, considering nutrient indices and microbial shifts, this enzyme increase may represent localized short-term effects (Ma et al., 2017).

### 4.2 Effects on soil microbial community and its functional diversity

Soil microorganisms are vital components of the soil ecosystem, playing crucial roles in promoting nutrient cycling and maintaining soil structure (Wang et al., 2023). PCoA of microbial community structure in this study clearly revealed distinct distribution patterns among samples from different treatments for both bacterial and fungal communities (Figures 1A,B). The microbial interaction networks were enhanced under all efficient cultivation patterns (Figures 3a–d; Table 3). The relative abundances of bacterial phyla often associated with nutrient cycling and soil health, including *Actinomycetota*, *Chloroflexota*, *Pseudomonadota*, and *Acidobacteriota* (Guo et al., 2024), were higher in the TM and TR treatments compared to the CK treatment (Figure 1C).

The TR treatment significantly increased the relative abundance of *Pseudomonadota* (+16.4%). *Pseudomonadota* is an exceptionally functionally diverse phylum, harboring numerous taxa involved in carbon and nitrogen cycling (e.g., denitrification, nitrogen fixation) and the rapid degradation of organic matter, particularly simple carbon sources (Zhang et al., 2022). Its enrichment showed significant positive correlations with the higher pH, elevated OM content, and significantly enhanced URE and SC activities observed in the TR treatment (Table 2; Figure 4). This indicates that the improved soil environment under this pattern provided favorable conditions for these bacteria with high metabolic activity, aligning with the findings of Li et al. (2020). The significant enrichment of *Mucoromycota* (+327%) and *Ascomycota* (+109%) in the fungal community contributed to a comprehensive improvement in soil fertility under the TR pattern. The TR treatment constructed the co-occurrence network with the highest number of nodes (107), the densest connections (edge number 2652), and the highest average degree (24.785), which was predominantly composed of positive interactions. Such a highly complex network structure typically signifies greater functional redundancy, resource utilization efficiency, and ecosystem stability (Chen et al., 2022; Hu et al., 2023; Li et al., 2024).

The TM treatment significantly enriched *Acidobacteriota* and *Chloroflexota* (+19.3% and +32.8%, respectively). *Acidobacteriota* is often associated with oligotrophic and acidic environments and is adept at decomposing recalcitrant organic matter, such as cellulose (de Castro et al., 2013; Hui et al., 2020). *Chloroflexota* exhibits similar traits, frequently participating in the anaerobic decomposition of complex OM (Xia and Xipeng, 2023). Furthermore, it showed a significant positive correlation with SC activity (Figure 4), which is consistent with the observed decrease in SC activity in TM (Xie et al., 2022). This may partially elucidate the observed phenomenon in TM of decreased SC activity alongside increased dehydrogenase activity. Dehydrogenases play a pivotal role in carbon metabolism by participating in redox reactions that facilitate the decomposition of OM and the release of energy (Blankinship et al., 2014; Hafez et al., 2022). By enhancing dehydrogenase activity, microorganisms can more efficiently decompose complex carbon sources, such as cellulose, hemicellulose, and lignin (Subi and Sheela, 2020; Xiong et al., 2021). Zhang B.-z. et al. (2023) found that CcpA regulates metabolic processes in response to varying glucose concentrations. Therefore, when simple carbon sources (e.g., sucrose) become limited, microorganisms may enhance dehydrogenase activity to decompose complex carbon sources, thereby sustaining their metabolic activities. The network complexity of TM (90 nodes, 1,625 edges) was higher than that of CK (Table 3) but lower than that of TR. Additionally, it exhibited higher modularity (0.492), indicating a structure composed of multiple relatively independent submodules. This suggests the potential for distinct resilience characteristics when facing perturbations (J et al., 2022).

The TP treatment exhibited significant pathogenic risks in the microbial community. *Olpidium bornovanu*s belongs to the genus *Olpidium* (phylum Olpidiomycota), a known pathogenic genus. At the fungal species level, the relative abundances of known or potential pathogens, such as *Olpidium bornovanus* and *Aspergillus fumigatus*, surged (+160% and +135%, respectively). Ziyu et al. (2021) found that phenolic acid allelochemicals are considered significant factors contributing to continuous cropping obstacles. Therefore, the enrichment of pathogens may be associated with the continuous secretion of phenolic acid allelochemicals (e.g., vanillic acid, trans-ferulic acid) from sweet potato roots. Within the bacterial community, *Nitrospirota* emerged as the most significantly enriched phylum (Figure 1C). Bacteria within this phylum dominate the nitrification process (ammonia oxidation to nitrite). Furthermore, the abundances of beneficial bacterial phyla, including *Acidobacteriota*, *Pseudomonadota*, *Chloroflexota*, and *Actinomycetota*, were generally and significantly reduced. This reflects a functional imbalance within the overall microbial community.

### 4.3 Effects of different cropping patterns on soil microbial functional diversity: molecular regulation of carbon and nitrogen metabolic pathways

Soil microbial functional diversity not only reflects soil biological activity but also characterizes the ecological traits of microbial communities (Zhu et al., 2023). Through metagenomic analysis, this study provides deep insights into the molecular regulatory mechanisms by which different cropping patterns influence the core metabolic functions of soil microorganisms—specifically carbon and nitrogen cycling—offering direct evidence for understanding the functional outputs of community compositional changes. Furthermore, at level 3, fundamental carbon metabolic pathways such as Glycolysis/Gluconeogenesis, ABC transporters, Pyruvate metabolism, and Oxidative phosphorylation exhibited high abundance (Figure 5a). This indicates robust overall metabolic activity of the soil microorganisms (Li et al., 2021). LEfSe analysis (Figure 5b) revealed that the CK treatment was notably enriched in fundamental amino acid metabolic pathways. The TP treatment showed distinct enrichment in carbohydrate metabolic pathways, including oxidative phosphorylation. The TR treatment exhibited significant enrichment in pathways like nitrogen metabolism. The TM treatment had fewer enriched pathways, primarily those related to xenobiotics biodegradation and metabolism and carbohydrate metabolism, such as chlorocyclohexane and chlorobenzene degradation. Li et al. (2019) proposed that under continuous cropping conditions, alterations occur in plant metabolic pathways, leading to impaired plant growth, changes in root distribution, and shifts in root exudates. In this study, the significant enrichment of amino acid metabolic pathways in the flue-cured CK treatment might be attributed to the fact that the duration of monocropping in Zhangjiajie has not exceeded 5 years; thus, the alteration of soil metabolic pathways has not yet reached a pronounced level (Yu et al., 2024). However, significant differences existed in the activity levels of core carbon metabolic pathways among the different treatments.

The TR treatment exhibited the highest abundance levels of key genes involved in glycolysis/gluconeogenesis (GAPDH, PGK) and the TCA cycle (aceE, korA) (Figure 6b). Notably, the abundance of aceE was increased by 34.7% and korA was significantly increased by 42.3% compared to CK. This is crucial for carbon flux and carbon fixation via the reductive TCA (rTCA) cycle (Gao et al., 2024). This highly efficient core carbon metabolic potential is intimately linked to the enriched Pseudomonadota (adept at utilizing soluble carbon sources for aerobic respiration) and the observed microbial community structure in the TR treatment (Xu et al., 2021; Silva et al., 2022). Furthermore, the abundant organic carbon inputs from straw incorporation provide sustained substrate supply for these microbial communities and their primarily governed glycolysis and TCA cycle pathways (Wang et al., 2020; Zhang Y. et al., 2023).

In the TM treatment, the abundance of the PK was reduced to the lowest level (Figure 6b). As PK is one of the three rate-limiting enzymes in glycolysis (Dong et al., 2022), this likely restricted glycolytic flux. Conversely, the abundance of the oorA gene (encoding 2-oxoglutarate ferredoxin oxidoreductase) was relatively high, which may promote α-ketoglutarate metabolism. This may be related to the specific metabolic capabilities of the enriched phyla in TM, *Acidobacteriota* and *Chloroflexota. Chloroflexota* can generate succinyl-CoA through oorA-mediatedα-ketoglutarate (α-KG) metabolism and further participate in sulfur metabolism, such as assimilatory sulfate reduction (Zheng et al., 2024). The TM pattern failed to exhibit the overall highly efficient carbon metabolic activity seen in TR. This is corroborated by its significantly reduced SC activity (Table 2). This reflects a potential shift in its carbon source utilization pathway towards complex polysaccharides or cellulose-like substances input from maize roots, rather than the glycolysis-dominated rapid pathway (Santangeli et al., 2024). This aligns with the findings of Niedeggen et al. (2024), who reported that maize root exudates are predominantly composed of high-molecular-weight polysaccharides, requiring microorganisms to expend more energy to activate degradation enzymes, resulting in lower carbon metabolic efficiency compared to systems dominated by glycolysis.

The TP treatment exhibited widespread reduction in the abundance of genes related to carbon metabolism. Abundance of key glycolysis genes (pfkA, PK) and the core carbon fixation gene (cbbL) was significantly reduced (Figure 6b). Notably, cbbL abundance plummeted by 94.5%, severely constraining the carbon fixation capacity of the Calvin cycle (Yin et al., 2022). Simultaneously, abundance of the accA gene (encoding propionyl-CoA carboxylase) was higher in TP, suggesting a metabolic flux shift towards the propionate synthesis pathway. This metabolic shift is closely associated with resource depletion due to the proliferation of pathogenic fungi (e.g., *Aspergillus fumigatus*) in TP, coupled with the broad inhibitory effects of phenolic acid allelochemicals on microorganisms. Consequently, microbial functions involved in fundamental carbon metabolism were impaired (Latgé and Chamilos, 2019).

Nitrogen availability is a critical determinant of agricultural productivity. Soil nitrogen transformation processes significantly influence soil nitrogen availability and nitrogen use efficiency (Deb et al., 2025). The results revealed that the ammonia assimilation pathway serves as the dominant route in nitrogen metabolism across all treatments (Figure 7a), and this finding aligns with the research of Zhao et al. (2024).

The TR treatment facilitated a highly efficient nitrogen cycling pattern. Not only did the gene abundances of glnA and gdh (gudB) peak (glnA increased by 19.6% compared to CK), but its ammonia oxidation process was also highly active. The abundances of key genes regulating ammonia monooxygenase (pmoA-amoA, pmoB-amoB, pmoC-amoC) were significantly higher than in other treatments. Specifically, pmoC–amoC abundance increased 4.6-fold compared to CK (Figure 7b). This aligns with findings by Jin et al. (2024) that TR significantly enhances the abundance of genes like nifH, amoA, and nirK, promoting the synergy between nitrification and nitrogen fixation, and improving soil ammonium nitrogen supply. This reflects intense ammonia oxidation under the TR pattern, supplying ample substrate (nitrite/nitrate) for subsequent efficient nitrogen assimilation (Wu et al., 2024). Simultaneously, the abundances of denitrification genes (nirK, nirS) were generally low (with nirK being the lowest among all treatments), significantly lower than in CK and TM. This “high-assimilation, low-denitrification” pattern (Figure 7a; Deb et al., 2025), effectively reduced nitrogen loss via gaseous forms (N_2_O, N_2_), promoting the conservation and recycling of nitrogen within the soil–microbe–plant system (Han et al., 2018; Yang et al., 2022). This efficient nitrogen cycling pattern was inextricably linked to the enriched *Pseudomonadota* phylum in TR, as well as the established complex and cooperative microbial network (Hestrin et al., 2019).

The significant enrichment of *Nitrospirota* in the TP treatment (Figure 1C) directly corresponds to its dominant role in nitrification, oxidizing ammonia to nitrite and subsequently to nitrate. However, the abundance of the nitrite reductase gene (nirS) was significantly reduced by 21.8% compared to CK (Figure 7b). This indicates that while nitrification potential is strong, the subsequent reduction of nitrite (to NO) potential is impaired, posing a clear risk of nitrite (NO2^−^) accumulation. This nitrogen cycle imbalance may stem from intercropping-induced shifts in carbon source utilization patterns (e.g., amino acid-dominated carbon sources), potentially suppressing denitrifier activity (Zhou et al., 2023). Nitrite accumulation may exert phytotoxic effects on certain plant roots, it also serves as a key precursor for the potent greenhouse gas nitrous oxide N_2_O, and nitrate derived from its further oxidation is more susceptible to leaching losses (Nie and Li, 2020).

The TM treatment showed fewer enriched nitrogen-related pathways in LEfSe analysis (Figure 5b). Metagenomic results revealed that the abundances of core ammonia assimilation genes (glnA, gudB) were comparable to CK (Figure 7b). Furthermore, no significant advantage was observed in the abundances of nitrification (pmo-amo) or denitrification (nirK, nirS) genes, this aligns with findings by Yang et al. (2025). Considering its microbial community was dominated by *Acidobacteriota* and *Chloroflexota*–phyla adapted to oligotrophic conditions and reliant on organic nitrogen mineralization (Chen et al., 2024, Gonçalves et al., 2024), along with significantly lower soil AN content compared to CK (Table 1). This suggests that the nitrogen cycling in TM likely emphasizes gradual mineralization of the existing soil organic nitrogen pool rather than efficient assimilation of newly input inorganic nitrogen as observed in TR. Nitrogen fixation genes (e.g., nifH) were not significantly enriched.

## 5 Conclusion

In paddy-upland tobacco planting systems, different high-efficiency cropping patterns, through the reciprocal influences of root exudates among crops or via rhizosphere resource inputs (e.g., straw return), can alter the soil microenvironment, reshape soil fertility and microbial communities, and induce pattern-specific differentiation in soil carbon and nitrogen metabolic functions.

TR, through the input of readily decomposable carbon sources (straw return), shapes a highly active microbial community with complex network structures, primarily dominated by *Pseudomonadota*. This community establishes a nitrogen cycling pattern characterized by “high assimilation and low denitrification,” possessing efficient genetic potential for carbon and nitrogen metabolism. It achieves a synergy between enhanced soil fertility and efficient nutrient cycling.

TM, with its input of complex carbon sources, selects for an oligotrophic microbial community mainly composed of *Acidobacteriota* and *Chloroflexota*. At the genetic level, its carbon metabolism shifts toward pathways for utilizing complex carbon sources, while nitrogen metabolism relies more heavily on the mineralization of organic nitrogen. Although this pattern can improve some available nutrients and form modular network structures, its glycolytic potential is constrained.

TP, due to the continuous input of phenolic allelochemicals, leads to the deterioration of the soil microecology. This is manifested by the enrichment of pathogenic bacteria, suppression of beneficial microbes, widespread inhibition of core carbon and nitrogen metabolic genes, and carries a genetic risk of nitrite accumulation.

## Data Availability

The datasets generated during and/or analyzed during the current study are available from the corresponding author on reasonable request. Raw shotgun metagenomic sequences in this study have been deposited in the National Center for Biotechnology Information (NCBI) under project accession PRJNA1331497.

## References

[ref1] Aleminew A. (2024). Enhancing soil fertility and crop productivity through crop residue management: a review. Agric. Rev. 45, 715–718. doi: 10.18805/ag.rf-294

[ref2] Blankinship J. C. Becerra C. A. Schaeffer S. M. Schimel J. P. (2014). Separating cellular metabolism from exoenzyme activity in soil organic matter decomposition. Soil Biol. Biochem. 71, 68–75. doi: 10.1016/j.soilbio.2014.01.010

[ref3] Chen X.-l. Guo T.-f. Zhang Q. Yue K. Zhang K.-k. Song X. . (2024). Microbial community succession of assimilating and utilizing straw-derived carbon based on DNA stable-isotope probing technique. J. Plant Nutr. Fertil. 30, 430–440. doi: 10.11674/zwyf.2023413

[ref4] Chen W. Wang J. Chen X. Meng Z. Xu R. Duoji D. . (2022). Soil microbial network complexity predicts ecosystem function along elevation gradients on the Tibetan plateau. Soil Biol. Biochem. 172:108766. doi: 10.1016/j.soilbio.2022.108766

[ref5] Chen J. Yang X. Zhong D. Huo Z. Sun R. Dong H. (2025). Continuous cropping alters soil microbial community assembly and co-occurrence network complexity in arid cotton fields. Agriculture 15:1274. doi: 10.3390/agriculture15121274

[ref6] Dai Y. Li J. Wang Z. Yang S. Xiao Q. Gao Z. . (2024). Effect of tobacco–radish rotation for different years on bacterial wilt and rhizosphere microbial communities. AMB Express 14:116. doi: 10.1186/s13568-024-01760-x, 39419902 PMC11486869

[ref7] de Castro V. H. L. Schroeder L. F. Quirino B. F. Kruger R. H. Barreto C. C. (2013). Acidobacteria from oligotrophic soil from the Cerrado can grow in a wide range of carbon source concentrations. Can. J. Microbiol. 59, 746–753. doi: 10.1139/cjm-2013-0331, 24206357

[ref8] Deb S. Espenberg M. Well R. Bucha M. Jakubiak M. Mander Ü. . (2025). Enhanced isotopic approach combined with microbiological analyses for more precise distinction of various N-transformation processes in contaminated aquifer – a groundwater incubation study. EGUsphere 2025, 1–35. doi: 10.5194/egusphere-2025-754

[ref9] Dong N. Chen L. Ahmad S. Cai Y. Duan Y. Li X. . (2022). Genome-wide analysis and functional characterization of pyruvate kinase (PK) gene family modulating rice yield and quality. Int. J. Mol. Sci. 23:15357. doi: 10.3390/ijms232315357, 36499684 PMC9739881

[ref9001] Gaind S. Singh Y. V. (2013). Soil Organic Carbon in Relation to Nutrient Regimes and Crop Rotation Under Rice-Based Cropping System. Journal of Crop Improvement 27, 170–185. doi: 10.1080/15427528.2012.746250

[ref10] Gao L. Liu L. Lv A. P. Fu L. Lian Z. H. Nunoura T. . (2024). Reversed oxidative TCA (roTCA) for carbon fixation by an Acidimicrobiia strain from a saline lake. ISME J. 18:wrae147. doi: 10.1093/ismejo/wrae147, 39073917 PMC11697166

[ref11] Gonçalves O. S. Fernandes A. S. Tupy S. M. Ferreira T. G. Almeida L. N. Creevey C. J. . (2024). Insights into plant interactions and the biogeochemical role of the globally widespread Acidobacteriota phylum. Soil Biol. Biochem. 192:109369. doi: 10.1016/j.soilbio.2024.109369

[ref12] Gong B. He Y. Luo Z. Peng H. Cai H. Zhu Y. . (2024). Response of rhizosphere soil physicochemical properties and microbial community structure to continuous cultivation of tobacco. Ann. Microbiol. 74:4. doi: 10.1186/s13213-023-01748-1

[ref51] Gu Y. Liu Y. Li J. Cao M. Wang Z. Li J. . (2021). Mechanism of intermittent deep tillage and different depths improving crop growth from the perspective of rhizosphere soil nutrients, root system architectures, bacterial communities, and functional profiles. Front. Microbiol. 12:759374. doi: 10.3389/fmicb.2021.759374, 35082764 PMC8784561

[ref13] Guo C. Yang C. Fu J. Song Y. Chen S. Li H. . (2024). Effects of crop rotation on sugar beet growth through improving soil physicochemical properties and microbiome. Ind. Crop. Prod. 212:118331. doi: 10.1016/j.indcrop.2024.118331

[ref9002] Hafez M. Ge S. Tsivka K. I. Popov A. I. Rashad M. (2022). Enhancing Calcareous and Saline-Sodic Soils Fertility by Increasing Organic Matter Decomposition and Enzyme Activities: An Incubation Study. Communications in Soil Science and Plant Analysis, 53, 2447–2459. doi: 10.1080/00103624.2022.2071930

[ref14] Han X. Shen W. Zhang J. Müller C. (2018). Microbial adaptation to long-term N supply prevents large responses in N dynamics and N losses of a subtropical forest. Sci. Total Environ. 626, 1175–1187. doi: 10.1016/j.scitotenv.2018.01.132, 29898524

[ref15] Hestrin R. Hammer E. C. Mueller C. W. Lehmann J. (2019). Synergies between mycorrhizal fungi and soil microbial communities increase plant nitrogen acquisition. Commun. Biol. 2:233. doi: 10.1038/s42003-019-0481-8, 31263777 PMC6588552

[ref16] Hiremath S. S. Prasanna N. L. S S. M A. K A. C. Nigam R. . (2024). A review on role of root exudates in shaping plant-microbe-pathogen interactions. J. Adv. Microbiol. 24, 1–17. doi: 10.9734/jamb/2024/v24i12868

[ref17] Hu J. Sun Y. Liu M. Zhao Y. Lv H. Wang Y. . (2023). Drip fertigation with straw incorporation promotes soil microbial network complexity and potentially reduces pathogen abundance in greenhouse vegetable production systems. Agric. Ecosyst. Environ. 351:108501. doi: 10.1016/j.agee.2023.108501

[ref55] Huang Y. B. Tang W. G. Xiao X. P. Tang H. M. Li C. Cheng K. K. . (2019). Effects of different planting patterns on the apparent balance of soil nutrients and nitrogen production efficiency in paddy soil. Ying Yong Sheng Tai Xue Bao = J. Appl. Ecol. 30, 1119–1126. doi: 10.13287/j.1001-9332.201904.024 30994271

[ref18] Hui C. Jiang H. Liu B. Wei R. Zhang Y. Zhang Q. . (2020). Chitin degradation and the temporary response of bacterial chitinolytic communities to chitin amendment in soil under different fertilization regimes. Sci. Total Environ. 705:136003. doi: 10.1016/j.scitotenv.2019.136003, 31846813

[ref19] J S. Y S. X W. J W. (2022). Microplastics reduce soil microbial network complexity and ecological deterministic selection. Environ. Microbiol. 24, 2157–2169. doi: 10.1111/1462-2920.15955 35229440

[ref20] Jin X. Yang X. Peng S. Ma E. Zhang H. Lin X. . (2024). Cropping rotation improved the bacterial diversity and N-cycling genes in tobacco fields through a 19-year long-term experiment. Appl. Soil Ecol. 193:105165. doi: 10.1016/j.apsoil.2023.105165

[ref21] Ke Z. Ling Y. Yong-jiang L. (2010). Effects of cropping patterns on yield and quality of flue-cured tobacco, soil nutrients and enzyme activities. J. Plant Nutr. Fertil. 16, 124–128. doi: 10.11674/zwyf.2010.0118

[ref22] Kunkun W. Tao R. Rihuan C. Zhifeng L. Xiaokun L. Jianwei L. (2024). Reduction of chemical phosphate fertilizer application in a rice–rapeseed cropping system through continuous straw return. Field Crop Res. 312:109399. doi: 10.1016/j.fcr.2024.109399

[ref23] Latgé J.-P. Chamilos G. (2019). *Aspergillus fumigatus* and Aspergillosis in 2019. Clin. Microbiol. Rev. 33:e00140-18. doi: 10.1128/cmr.00140-00118, 31722890 PMC6860006

[ref24] Li J. Chen X. Zhan R. He R. (2019). Transcriptome profiling reveals metabolic alteration in *Andrographis paniculata* in response to continuous cropping. Ind. Crop. Prod. 137, 585–596. doi: 10.1016/j.indcrop.2019.05.067

[ref25] Li J. Gao K. Wan L. Cao G. Jiao F. Wang Y. . (2020). Effects of microbial agent on the growth of *Catalpa bungei* seedlings and the diversity of bacterial community in rhizosphere soil. Acta Ecol. Sin. 40, 7588–7601. doi: 10.5846/stxb201901030031

[ref26] Li Z. Han J. Bai H. Peng D. Wang L. Bai L. (2021). Effects of novel bioorganic fertilizer application on soil enzymes and bacterial community in multi-site rice paddies in China. AMB Express 11:79. doi: 10.1186/s13568-021-01241-5, 34057636 PMC8167081

[ref27] Li W. Shi F. Yi S. Feng T. Wang C. Li Z. . (2024). Soil multifunctionality predicted by bacterial network complexity explains differences in wheat productivity induced by fertilization management. Eur. J. Agron. 153:127058. doi: 10.1016/j.eja.2023.127058

[ref28] Liang-liang M. A. O. Xi-bin D. Hang-feng Q. U. Bao-shan Z. Hui L. I. U. Ran G. A. O. . (2023). Effects of thinning on soil microbial enzyme activity in natural conifer and broadleaved mixed forest in Xiaoxing’an mountains. For. Eng. 39, 36–45. Available online at: https://www.aeeisp.com/slgc/en/article/id/8f6881b7-2ea5-4718-9761-559b7a8bc537

[ref29] Ma Y.-h. Fu S.-l. Zhang X.-p. Zhao K. Chen H. Y. H. (2017). Intercropping improves soil nutrient availability, soil enzyme activity and tea quantity and quality. Appl. Soil Ecol. 119, 171–178. doi: 10.1016/j.apsoil.2017.06.028

[ref30] Majumder S. Powell M. A. Biswas P. K. Banik P. (2022). The impact of arsenic induced stress on soil enzyme activity in different rice agroecosystems. Environ. Technol. Innov. 26:102282. doi: 10.1016/j.eti.2022.102282

[ref32] Nie M. Li Z. (2020). Bioprocess of nitrite accumulation in water - a review. Sheng Wu Gong Cheng Xue Bao 36, 1493–1503. doi: 10.13345/j.cjb.190578, 32924348

[ref33] Niedeggen D. Rüger L. Oburger E. Santangeli M. Ahmed M. Vetterlein D. . (2024). Microbial utilisation of maize rhizodeposits applied to agricultural soil at a range of concentrations. Eur. J. Soil Sci. 75:e13530. doi: 10.1111/ejss.13530

[ref34] Olougou M. N. E. Achiri D. T. Ngone M. A. Ndzeshala S. D. Tchakounté G. V. T. Tening A. S. . (2024). Bio-inoculant consortia modulated plantain (*Musa × paradisiaca* L.) growth, rhizosphere pH, acid phosphatase and urease activity. Soil Adv. 2:100008. doi: 10.1016/j.soilad.2024.100008

[ref38] Santangeli M. Steininger-Mairinger T. Vetterlein D. Hann S. Oburger E. (2024). Maize (*Zea mays* L.) root exudation profiles change in quality and quantity during plant development – a field study. Plant Sci. 338:111896. doi: 10.1016/j.plantsci.2023.111896 37838155

[ref39] Silva G. d. C. Kitano I. T. Ribeiro I. A. d. F. Lacava P. T. (2022). The potential use of actinomycetes as microbial inoculants and biopesticides in agriculture. Front. Soil Sci. 2:833181. doi: 10.3389/fsoil.2022.833181

[ref40] Soil Science Society of America Weaver R. W. (1994). Methods of Soil Analysis. Part 2, Microbiological and Biochemical Properties. Madison, WI: Soil Science Society of America.

[ref41] Subi S. Sheela A. M. (2020). Microbial activity and cellulose degraders in termite mound soil. Int. J. Curr. Microbiol. Appl. Sci. 9, 2154–2161. doi: 10.20546/ijcmas.2020.907.251

[ref42] Sun W. Li Q. Qiao B. Jia K. Li C. Zhao C. (2024). Advances in plant–soil feedback driven by root exudates in forest ecosystems. Forests 15:515. doi: 10.3390/f15030515

[ref35] Sun Q. Wu H. L. Chen F. Kang J. H. (2020). Characteristics of soil nutrients and fungal community composition in crop rhizosphere under different rotation patterns. Huan jing ke xue= Huanjing kexue 41, 4682–4689. doi: 10.13227/j.hjkx.202001031 33124401

[ref44] Tamburini G. Bommarco R. Wanger T. C. Kremen C. van der Heijden M. G. A. Liebman M. . (2020). Agricultural diversification promotes multiple ecosystem services without compromising yield. Sci. Adv. 6:eaba1715. doi: 10.1126/sciadv.aba1715, 33148637 PMC7673676

[ref45] Wang Z. Guo X. Cao S. Yang M. Gao Q. Zong H. . (2024). Tobacco/Isatis intercropping system improves soil quality and increase total production value. Front. Plant Sci. 15:1458342. doi: 10.3389/fpls.2024.1458342, 39624241 PMC11608962

[ref37] Wang S. Heal K. V. Zhang Q. Yu Y. Tigabu M. Huang S. . (2023). Soil microbial community, dissolved organic matter and nutrient cycling interactions change along an elevation gradient in subtropical China. J. Environ. Manag. 345:118793. doi: 10.1016/j.jenvman.2023.118793 37619380

[ref46] Wang J. Ma L. J. Long Z. H. Min W. Hou Z. A. (2020). Effects of straw biochar on soil microbial metabolism and bacterial community composition in drip-irrigated cotton field. Huan Jing Ke Xue 41, 420–429. doi: 10.13227/j.hjkx.201907183, 31854945

[ref47] Wu T. Gu G. Zhang B. Zhang Z. Chi G. Wang R. . (2024). Physicochemical properties and microbial community of soil and crop yield under rice-tobacco-milk vetch rotation cropping. Fujian J. Agric. Sci. 39, 984–992. doi: 10.19303/j.issn.1008-0384.2024.08.012

[ref48] Xia W. Xipeng J. (2023). Effect of different continuous cropping years on the rhizosphere soil microbial community structure of *Tussilago farfara* L. Res. Sq. doi: 10.21203/rs.3.rs-3715731/v1

[ref49] Xie K. Sun M. Shi A. Di Q. Chen R. Jin D. . (2022). The application of tomato plant residue compost and plant growth-promoting rhizobacteria improves soil quality and enhances the ginger field soil bacterial community. Agronomy 12:1741. doi: 10.3390/agronomy12081741

[ref36] Xiong Q. Qiao J. Wang M. Li S. Li X. (2021). Carboxylated and quaternized lignin enhanced enzymatic hydrolysis of lignocellulose treated by p-toluenesulfonic acid due to improving enzyme activity. Bioresour. Technol. 337:125465. doi: 10.1016/j.biortech.2021.125465 34320745

[ref50] Xu Z. Chen X. Wei Y. Zhang Q. Ji X. (2021). Metagenomic analysis of the diversity of microbes in the Napahai plateau wetland and their carbon and nitrogen metabolisms. Sheng Wu Gong Cheng Xue Bao 37, 3276–3292. doi: 10.13345/j.cjb.200658, 34622635

[ref52] Yang M. Zhao J. Yuan Y. Chen X. Yang F. Li X. (2021). Comparative metagenomic discovery of the dynamic cellulose-degrading process from a synergistic cellulolytic microbiota. Cellulose 28, 2105–2123. doi: 10.1007/s10570-020-03671-z

[ref53] Yang X. Zhu Y. Xu Y. Li X. Zhang S. Qian Q. . (2022). Simulated warming and low O_2_ promote N_2_O and N_2_ emissions in subtropical montane forest soil. J. Soils Sediments 22, 2706–2719. doi: 10.1007/s11368-022-03234-8

[ref54] Yang K. Zi S. Ouyang C. (2025). Effects of the tobacco-maize relay intercropping pattern on soil nutrients and soil microbial diversity. Front. Microbiol. 15:1389156. doi: 10.3389/fmicb.2024.1389156, 39867492 PMC11757017

[ref56] Yifan T. Chengren O. Ge W. Tao L. Kai L. Shuhui Z. (2024). Influences of interplanting corn with flue-cured tobacco on soil nutrient supply and antioxidant properties of tobacco plants. Jiangsu Agric. Sci. 52, 99–104. doi: 10.15889/j.issn.1002-1302.2024.01.014

[ref57] Yin T. Qin H.-l. Yan C.-r. Liu Q. He W.-q. (2022). Low soil carbon saturation deficit limits the abundance of cbbL-carrying bacteria under long-term no-tillage maize cultivation in northern China. J. Integr. Agric. 21, 2399–2412. doi: 10.1016/S2095-3119(21)63800-5

[ref58] Yu T. Hou X. Fang X. Razavi B. Zang H. Zeng Z. . (2024). Short-term continuous monocropping reduces peanut yield mainly via altering soil enzyme activity and fungal community. Environ. Res. 245:117977. doi: 10.1016/j.envres.2023.117977, 38141923

[ref59] Zhang B.-z. Borjigin Q. Gao J.-l. Yu X.-f. Hu S.-p. Wang F.-g. . (2023). Metagenomic analysis of microbial consortium GF-20 in corn stover degradation at low temperature. J. Environ. Eng. Landsc. Manag. 31, 92–102. doi: 10.3846/jeelm.2023.18489

[ref60] Zhang X. Huang Z. Zhong Z. Li Q. Bian F. Gao G. . (2022). Evaluating the rhizosphere and endophytic microbiomes of a bamboo plant in response to the long-term application of heavy organic amendment. Plants 11:2129. doi: 10.3390/plants11162129, 36015431 PMC9412275

[ref61] Zhang Y. Xiao F. Zhang L. Ding Z. Shi G. Li Y. (2023). A new mechanism of carbon metabolism and acetic acid balance regulated by CcpA. Microorganisms 11:2303. doi: 10.3390/microorganisms11092303, 37764147 PMC10535407

[ref62] Zhao Y. Zhu-Barker X. Cai K. Wang S. Wright A. L. Jiang X. (2024). Quest for the nitrogen-metabolic versatility of microorganisms in soil and marine ecosystems. Microorganisms 12:1283. doi: 10.3390/microorganisms12071283, 39065052 PMC11278940

[ref63] Zheng R. Wang C. Sun C. (2024). Deep-sea in situ and laboratory multi-omics provide insights into the sulfur assimilation of a deep-sea Chloroflexota bacterium. MBio 15, e00004–e00024. doi: 10.1128/mbio.00004-24, 38417116 PMC11005417

[ref64] Zhou M. Sun C. Dai B. He Y. Zhong J. (2023). Intercropping system modulated soil–microbe interactions that enhanced the growth and quality of flue-cured tobacco by improving rhizospheric soil nutrients, microbial structure, and enzymatic activities. Front. Plant Sci. 14:1233464. doi: 10.3389/fpls.2023.1233464, 37941660 PMC10628710

[ref65] Zhu B. Gu H. He J. Li F. Yu J. Liu W. . (2023). The impact of smash-ridge tillage on agronomic traits of tobacco plants, soil enzymatic activity, microbial community structure, and functional diversity. Plant Signal. Behav. 18:2260640. doi: 10.1080/15592324.2023.2260640, 37877306 PMC10730138

[ref66] Ziyu W. LiQiao L. Yueyue L. jialing J. Yong J. Min L. (2021). Accumulation and secretion of pinellitic acid allelochemicals in *Pinellia ternata* (Thunb.) Breit. Asian J. Ecotoxicol. 16, 335–344. doi: 10.7524/AJE.1673-5897.20201216001

